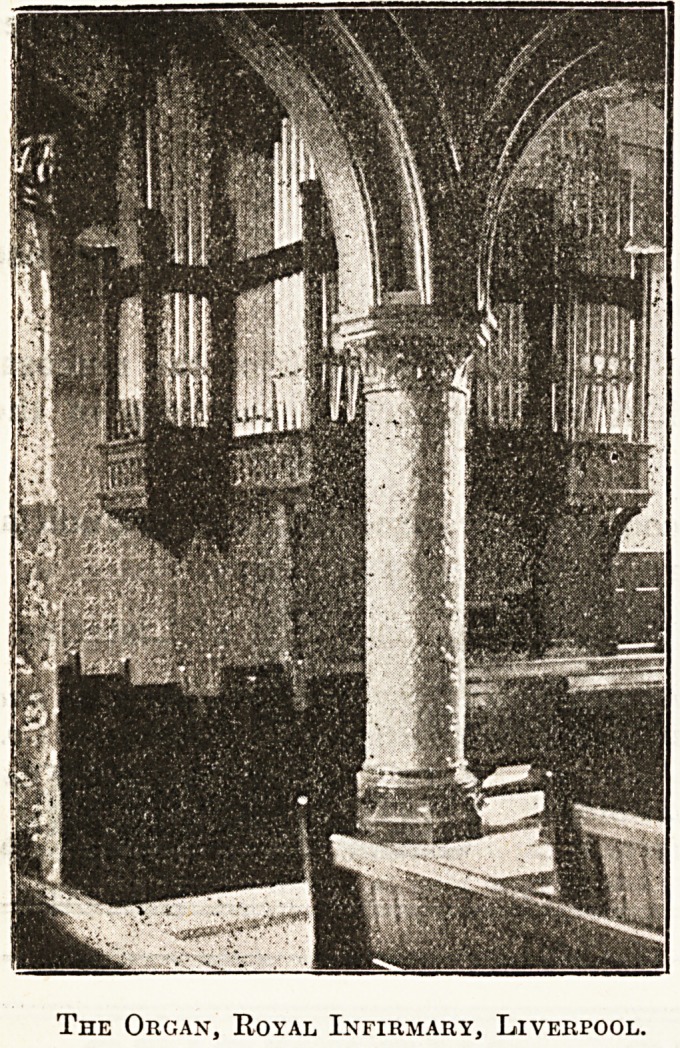# Royal Infirmary, Liverpool

**Published:** 1915-02-20

**Authors:** 


### IV.—SOME PROVINCIAL HOSPITAL CHAPELS.*

(Contributions for this Section Cordially Invited.)

### Royal Infirmary, Liverpool.

The chapel of the Royal Infirmary, Liverpool, accommodating 200 persons, was erected in the years 1887-1890, when the entire hospital was rebuilt according to the designs of the late Alfred Waterhouse, R.A. The architect, who was given a free hand in the decoration of the chapel, produced a design at once original and appropriate.

The Chapel of the Royal Infirmary, Liverpool.

The building consists of a nave and aisles, single on the south and double on the north side, where the organ is placed. The sanctuary is apsidal in shape, the ceiling is flat and divided into compartments corresponding with the bays, and the walls, arches, and pillars are all covered with faience of beautiful design in a soft bluish-green colouring, which is restful and in harmonious contrast with the woodwork of the seats and other furniture, which are of polished pitch pine. This work was executed by Messrs. Burmantofts, of Leeds, and is not only a fine example of this style of decoration, but one truly "hospital" in character in its asepticity, in keeping with the glazed brick walls of the hospital throughout. The windows of the chapel are round-headed and fitted with pleasing cathedral glass. The arches are of varied design, some being round and others slightly pointed; the planning is, however, very appropriate,

Another View of the Chapel.

* Previous articles appeared on January 23 and 30 and February 13.

because it carries out the scheme upon which the whole hospital is designed. The organ, which since its first erection has been enriched by various special gifts, is now an instrument of three manuals, with detached console pneumatically attached; it is entirely suited to the building, being for its size remarkably rich in varied soft effects.

Many memorials in brass adorn the walls, witnessing to

The Organ, Royal Infirmary, Liverpool.

faithful work in the service of the hospital, among them one in the chancel commemorating the forty-nine years' work of the late Chaplain, the Rev. W. Smith. The Rev. J. R. Darbyshire is the Chaplain to the institution.

## Figures and Tables

**Figure f1:**
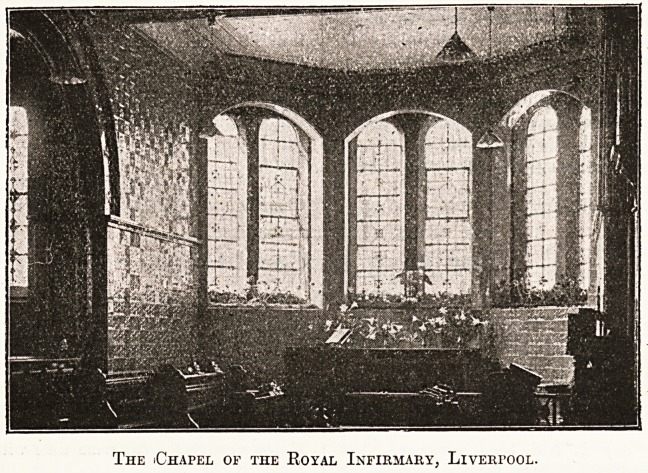


**Figure f2:**
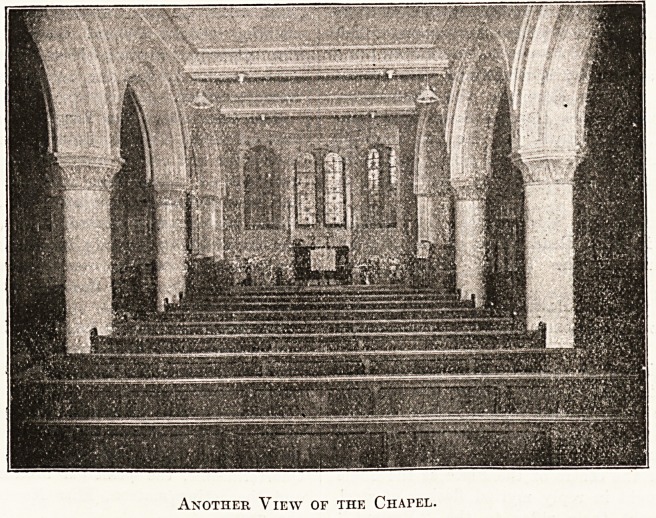


**Figure f3:**